# Separation of Flavonoids and Purification of Chlorogenic Acid from Bamboo Leaves Extraction Residues by Combination of Macroporous Resin and High-Speed Counter-Current Chromatography

**DOI:** 10.3390/molecules28114443

**Published:** 2023-05-30

**Authors:** Yifeng Zhou, Meixu Chen, Xinyi Huo, Qilin Xu, Linlin Wu, Liling Wang

**Affiliations:** 1 School of Biological and Chemical Engineering, Zhejiang University of Science and Technology, Hangzhou 310023, China; 2 Zhejiang Academy of Forestry, Hangzhou 310023, China

**Keywords:** bamboo leaves, flavonoids, macroporous resin, high-speed countercurrent chromatography (HSCCC), chlorogenic acid (CA)

## Abstract

Flavonoids are major active small-molecule compounds in bamboo leaves, which can be easily obtained from the bamboo leaves extraction residues (BLER) after the polysaccharides extraction. Six macroporous resins with different properties were screened to prepare and enrich isoorientin (IOR), orientin (OR), vitexin (VI), and isovitexin (IVI) from BLER, and the XAD-7HP resin with the best adsorption and desorption performance was selected for further evaluation. Based on the static adsorption experiments, the experimental results showed that the adsorption isotherm fitted well with the Langmuir isotherm model, and the adsorption process was better explained by the pseudo-second-order kinetic model. After the dynamic trial of resin column chromatography, 20 bed volume (BV) of upload sample and 60% ethanol as eluting solvent was used in a lab scale-up separation, and the results demonstrated that the content of four flavonoids could be increased by 4.5-fold, with recoveries between 72.86 and 88.21%. In addition, chlorogenic acid (CA) with purity of 95.1% was obtained in water-eluted parts during dynamic resin separation and further purified by high-speed countercurrent chromatography (HSCCC). In conclusion, this rapid and efficient method can provide a reference to utilize BLER to produce high-value-added food and pharmaceutical products.

## 1. Introduction

*Phyllostachys heterocycla* (Carr.) Mitford cv. Pubescens is the most widely distributed bamboo species in China. Bamboo leaves have a long history of being used as traditional Chinese medicine (TCM) and functional food. In TCM, it was documented as a sweet-smelling, flat-shaped cold drug, which was mainly used for the treatment of feverish chest and cough; it was also recorded to have diuretic, eyesight improvement, and detoxification properties [1]. Flavonoids are important natural active substances in bamboo leaves and have many biological and physiological functions, such as antibacterial, antioxidant, anti-free-radical, anti-aging, anti-tumor, and cardiovascular protection [1,2,3,4,5]. The active constituents of flavonoid and phenolic acid extracts from bamboo leaf include orientin (OR), isoorientin (IOR), isovitexin (IVI), vitexin (VI) (Figure S1), and chlorogenic acid (CA), among others. Several methods have been developed to separate flavonoids and phenolic acids from bamboo leaf, including zirconia adsorption [6], macroporous resin [7], homogenate-assisted vacuum-powered bubble extraction [8], mechanochemic-assisted extraction [9], mesoporous carbon adsorption [10], and ionic liquid extraction [11]. We speculated that there would be a large number of flavonoids in the alcohol precipitation after the water extraction of polysaccharides from bamboo leaves. However, this BLER also contains a large number of impurities, such as vitamins, amino acids, proteins, peptides, and polysaccharides [12,13,14], which inevitably increases the burden of repurifying to obtain flavonoids, because these components have similar polarity, solubility, and other properties with the target flavonoids. However, it is expected that more and more efficient methods for selective extraction and enrichment will provide new options for researchers.

Macroporous resin is a kind of commercial separation material with the advantages of low price, simple operation, high efficiency, minor pollution, and easy regeneration [15]. At present, natural products such as saponins [15], flavones [16,17], and alkaloids [18] are enriched and separated by macroporous resins, so it is theoretically feasible to use macroporous resins for the separation of flavonoids from bamboo leaves. Although macroporous resin absorption is a common method to separate flavonoid compounds from bamboo leaf extracts [19,20], phenolic acids (such as CA) are lost in the process of water washing to remove water-soluble impurities due to the weak interaction between phenolic acid and resin [7]. Therefore, it is of great significance to explore the separation method of CA to improve the added value of bamboo leaf products. HSCCC is a kind of chromatographic separation technology based on liquid-liquid distribution, without the need of stationary phase. Compared with traditional chromatographic technology, it has many unique advantages [21]. It can prevent sample loss caused by irreversible adsorption and can be directly applied to the crude extract, which has the characteristics of continuous high efficiency, high recovery, low solvent consumption, and batch production. Previously, macroporous resins have been used in combined with HSCCC to separate other components, such as saponins [22], lignans [23], anthraquinone [24], pelargonidin-3-O-glucoside [25], zearalenone [26], antioxidants [27], and so on. Occasionally, HSCCC or macroporous resin alone could not yield satisfactory results; therefore, a combination of two chromatographic methods might be required to achieve the separation of different target compounds. Thus, we speculated that HSCCC combined with macroporous resin can isolate flavonoids and CA from BLER.

In this study, the static and dynamic adsorption properties of four flavonoids from BLER were systematically investigated based on the chosen macroporous resin. At the same time, the purification conditions of CA from water elution fraction by HSCCC process were explored by resin dynamic adsorption. Our study will develop an effective method for the enrichment and separation of active ingredients from BLER and other bamboo leaf extracts, and also provide a reference for the separation and preparation of natural active substances from plant extracts using macroporous resin and HSCCC.

## 2. Results and Discussion

### 2.1. Resin Screening for Separation of the Flavonoids

**Figure 1:**
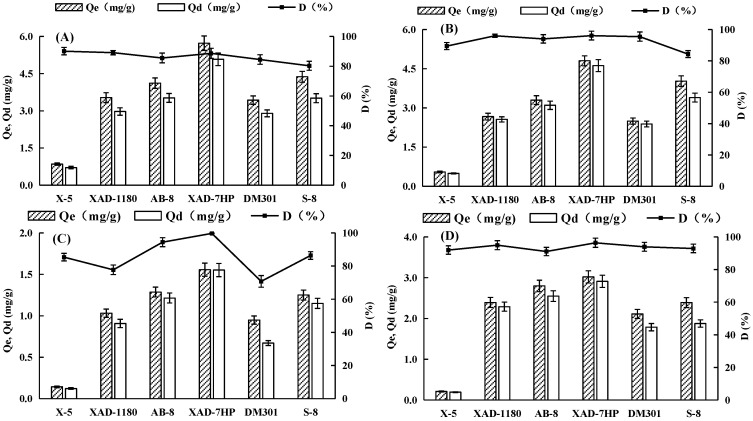
Adsorption capacities and desorption ratios of four flavonoids on six resins: IOR (**A**), OR (**B)**, VI (**C**), and IVI (**D**).

Previous studies have indicated that flavonoids can be adsorbed and separated by different macroporous resins [16,28,29,30,31], which is not only related to the characteristics of the extract solutions but also to the physical properties of the macroporous resins. The adsorption effect of the non-polar or weak polar resins on flavonoids was better than that of the polar resins, indicating that the flavonoids are weak polar compounds according to the polarity matching principle [32]. Therefore, six different resins, with a focus on those that are non-polar or have weak polarity (Table 1), were tested for their adsorption and desorption capacities for four flavonoids in BLER (Figure 1).

As shown in Figure 1, the adsorption capacities of four flavonoids are ranked as follows: IOR > OR > IVI > VI, which is consistent with their concentrations in the BLER solution. The adsorption capacity of XAD-7HP on the four flavonoids was better than that of the other five resins, and its maximum average pore diameter may be the key factor affecting the adsorption of flavonoids in BLER solution. In desorption capacity tests, all these resins displayed almost equivalent desorption ratios (>80%) except for VI, which also suggested that these resins can be used for BLER and regenerated efficiently. Therefore, the XAD-7HP resin was chosen for further studies.

### 2.2. Adsorption Isotherms of the Flavonoids on XAD-7HP Resin

**Figure 2:**
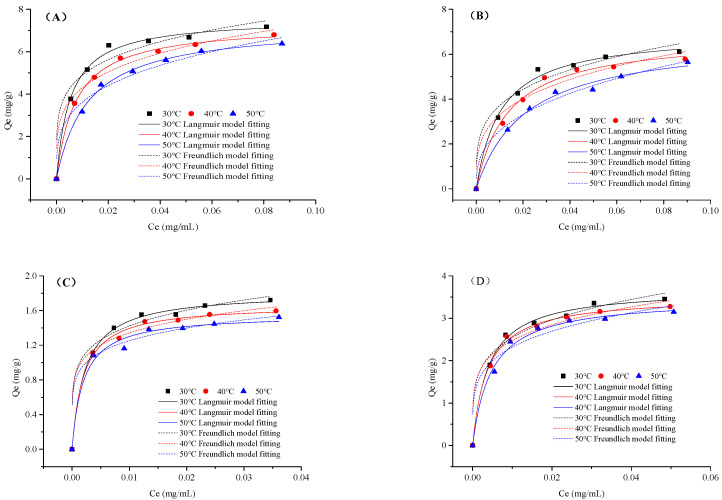
Adsorption isotherms of different flavonoids on XAD-7HP resin at different temperatures (30 °C, 40 °C, 50 °C): IOR (**A**), OR (**B**), VI (**C**), and IVI (**D**).

Figure 2 shows the adsorption isotherms of four flavonoids from BLER aqueous solutions on XAD-7HP resin at different temperatures (30 °C, 40 °C, 50 °C). At the relatively lower equilibrium concentration, the adsorption capacity of XAD-7HP resin on four flavonoids increased significantly, but its capacity leveled off with the increase of equilibrium concentrations. Moreover, the adsorption capacity of four flavonoids decreased with the increase of temperatures. Considering the efficiency and solubility, the initial concentration of BLER was selected as 20 mg/mL and the temperature as 30 °C for subsequent experiments.

Fitting with classic isotherm models, the parameters from the Langmuir and Freundlich equations are summarized in Table 2. The results showed that the Langmuir equation could well describe the adsorption of four flavonoids on XAD-7HP resin and had good correlation coefficients (*R*^2^), which suggested that the adsorption process of four flavonoids was mainly monolayer solid-liquid adsorption [33]. Generally, in the Freundlich equation, the adsorption easily took place when the 1/*n* value was between 0.1 and 0.5; it tended not to happen when the 1/*n* value was between 0.5 and 1.0, and it was almost unable to occur when the 1/*n* value exceeded 1 [32]. Table 3 shows that the values of 1/*n* for four flavonoids were between 0.1 and 0.5, which indicates that adsorption on the resin could take place easily and the adsorption processes were good.

### 2.3. Adsorption Kinetics of the Flavonoids on XAD-7HP Resin

**Figure 3:**
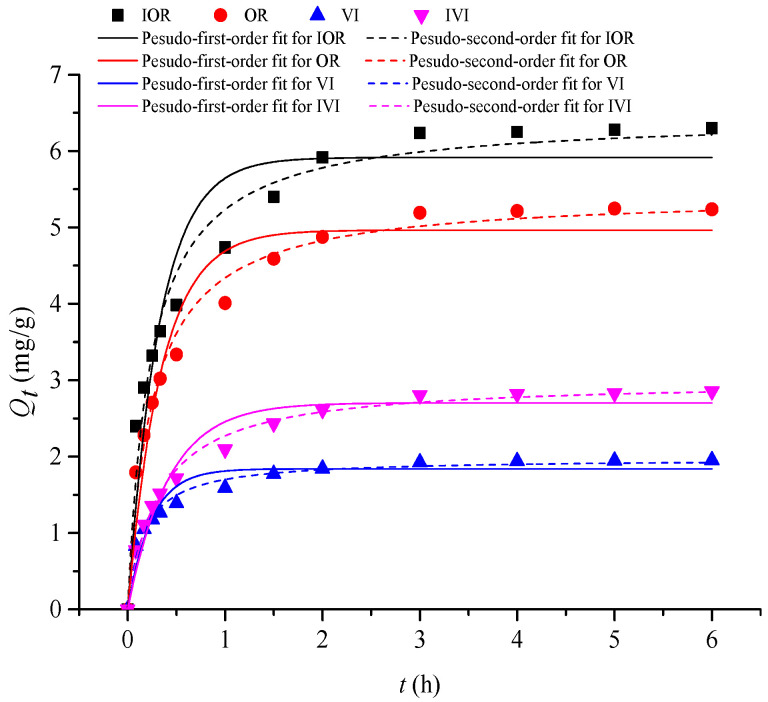
Adsorption kinetic curves of flavonoids on the XAD-7HP resin at 30 °C.

As shown in Figure 3, the uptake amounts of the flavonoids exhibited a rapid increase followed by a leveling-off process. The results showed that XAD-7HP resin exhibited a highly efficient adsorption on the four flavonoids, and the adsorption rate reached 80% within 1.5 h and reached equilibrium within 6 h. In Table 3, the higher correlation coefficients (*R*^2^) indicated that the pseudo-second-order rate equation is more suitable to analyze the adsorption kinetics of four flavonoids on the XAD-7HP than the pseudo-first-order rate equation; moreover, the theoretical values of *Q_e_* in the pseudo-second-order rate equation are very close to the experimental results. These results indicated that the adsorption process of the flavonoids is more like a second-order process, with a fast initial step followed by a slower second step, which is controlled by pore diffusion or a slow adsorption reaction [34].

### 2.4. Dynamic Adsorption and Desorption of the Flavonoids on XAD-7HP Resin

**Figure 4:**
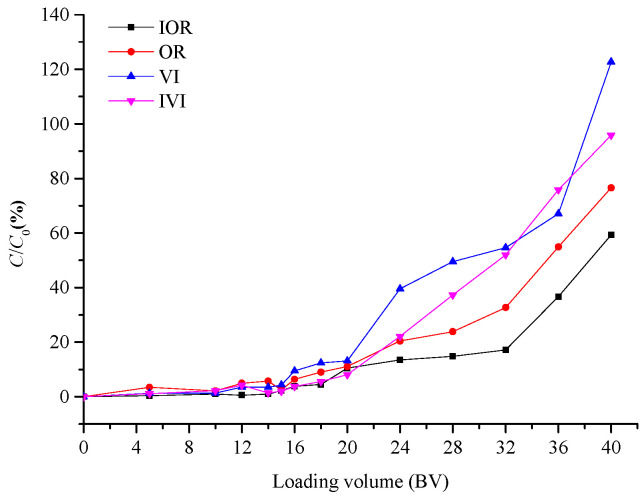
Dynamic breakthrough curves for four flavonoids.

In general, the breakthrough points are defined as the effluent volume when the effluent sample concentration reaches 5% of the initial concentration [28]. As shown in Figure 4, the breakthrough volume of OR and VI on the XAD-7HP resin was 16 BV, while the breakthrough volume of IOR and IVI was 18 BV. Then, the concentration of four flavonoids in the effluent increased gradually, reaching about 10% of the initial concentration at 20 BV. Thinking about saving the dosage of resin, the sample solution of 20 BV was considered as the appropriate sample volume on the XAD-7HP resin column.

**Figure 5:**
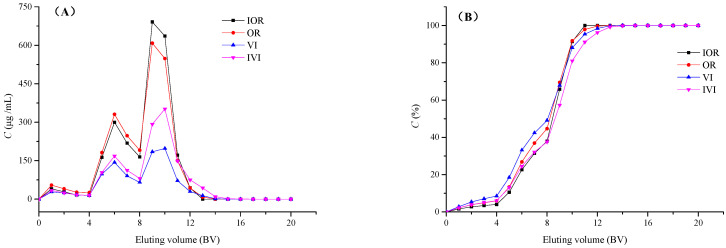
Dynamic desorption curves for four flavonoids: effluent concentration (**A**) and the cumulative relative desorption ratio (**B**).

Appropriate desorption concentration is an important factor in the dynamic desorption process [35]. As shown in Figure 5A, the concentration of four flavonoids was relatively small at the initial 4 BV in 20% ethanol effluent and increased gradually with 40% ethanol effluent (5–8 BV). The 60% ethanol effluent (9–12 BV) reached the highest concentration and decreased sharply to the lowest value when eluted by 80% ethanol (13–16 BV) and 100% ethanol (17–20 BV). Figure 5B shows the cumulative relative desorption ratio (%) of the four flavonoids on the XAD-7HP resin. After eluting with 60% ethanol, the values were all greater than 98%, indicating that 60% ethanol was an appropriate concentration for the dynamic desorption process.

In addition, a high concentration of CA was found unexpectedly in the effluent of the loading sample and the water eluting process. Therefore, the effluent from these two fractions was collected to prepare high-purity CA by HSCCC in the subsequent test.

### 2.5. Lab Scale-Up Separation of the Flavonoids on XAD-7HP Resin

Figure S2 presented the HPLC chromatograms of different eluting fractions of XAD-7HP in the scale-up dynamic enrichment experiment. In the scale-up dynamic enrichment experiment, the content of four flavonoids was increased by XAP-7HP, and the recovery was acceptable; the content of IOR, OR, VI, and IVI increased to 1.63%, 1.56%, 0.53%, and 0.86% with recoveries of 88.21%, 86.79%, 74.72%, and 72.86%, respectively. These experimental results are accorded with those of the previous trial’s dynamic tests. In addition, the content of CA in the water eluting fraction increased from 1.17% to 5.48%.

### 2.6. Purification of CA by HSCCC

For HSCCC separation, a suitable two-phase solvent system is crucial, which requires an appropriate partition coefficient and good sample solubility [36]. Generally, the most suitable range of K values for HSCCC separation is 0.5–2 [37]. As shown in Table 4, according to the values of K, ethyl acetate/acetonitrile/acetic acid/water (*v*/*v*, 7:2:1:10) was chosen as the solvent system of HSCCC.

Figure S3 shows the HSCCC separation chromatogram of CA within 280 min, and the effluent fractions were analyzed by HPLC according to collection program. On the basis of the HPLC analysis, CA (20 mg) was obtained in the fraction within 140–160 min with purity of 95.1% (Figure S4).

## 3. Materials and Methods

### 3.1. Chemicals and Reagents

Six different types of macroporous resins were bought from H&E Co., Ltd. (Beijing, China). The chemical standards used for HPLC content analysis or HSCCC compound identification were obtained from Chengdu Pure Chem-Standard Co., Ltd. (Chendu, China). HPLC-grade acetonitrile was purchased from Aladdin Industrial Corporation (Shanghai, China). The pure water was prepared by a Milli-Q^®^ Direct Water Purification System from Millipore Corporation (Bedford, MA, USA). Other analytical-grade solvents used in this study were produced by Lingfeng Chemical Reagent Company (Shanghai, China).

### 3.2. Apparatus

HPLC was performed on a Shimadzu LC-20AD separations module connected to a SIL-20A autosampler, a CTO-20A column oven, and an SPD-20AUV/visible detector (Shimadzu Corporation, Kyoto, Japan). A constant temperature shaker (Shanghai Tiancheng Experimental Instrument Manufacuring Co., Ltd., Shanghai, China) was used for static adsorption of macroporous resins. The HSCCC systems were comprised of a TBE-300A module, a TBP5002 constant-flow pump, and an ultraviolet (UV) monitor (TautoBiotechnique Co., Ltd., Shanghai, China). The TBE-300 Amodule was equipped with three polytetrafluoroethylene preparative coils (2.6 mm, i.d.; total volume, 300 mL) and a 20 mL sample injection loop, and the revolution speed could be adjusted in the range from 0 to 1000 rpm. A constant temperature regulator (HX-105, Beijing Changliu Science Implement, Beijing, China) was used to control the temperature of the separation coils. A CBS-A program-controlled automatic fraction collector (Shanghai Huxi analysis instrument factory Co., Ltd., Shanghai, China) was applied for collecting the effluent from HSCCC.

### 3.3. Preparation of Sample

The bamboo leaves were collected in Anji country (Huzhou City, Zhejiang Province, China), washed, air-dried, and stored at room temperature until use. The crude polysaccharides from bamboo leaves were prepared on the basis of the previous method [13], and then the discarded supernatant of ethanol solution was filtered, concentrated by rotary evaporator, and dried by vacuum freezing dryer to obtain the bamboo leaves extract residues (BLER).

### 3.4. Separation of Flavonoids by Macroporous Resin

#### 3.4.1. HPLC Determination of Flavonoids in Resin Separation

The determination method was performed according to the reference [38] with slight modification. Briefly, HPLC analysis was carried out on a Shimadzu WondaCract C18 chromatographic column (4.6 mm × 250 mm, 5 μm) at a column temperature of 35 °C. The mobile phase consisted of acetonitrile (solvent A) and 0.1% formic acid solution (solvent B), and the gradient elution was as follows: 0–15 min, A maintained at 15%; 15–30 min, A increased from 15% to 30%; 30–36 min, A increased to 80% for cleaning the column impurities; 36–42 min, A returned to 15% to achieve chromatography equilibrium for the next sample. The flow rate was maintained at 1.0 mL/min, and the detection wavelength was set at 325 nm.

In order to investigate the separation performance comprehensively, four flavonoids and four phenolic acids were simultaneously analyzed through chromatography, and the concentration of four flavonoids, IOR, OR, VI, and IVI, were determined by the calibration curves, where Y is the peak area of the target analyte and X is the concentration (μg/mL) of the four flavonoids, respectively. The linear correlation ranges of IOR, OR, VI, and IVI were found to be within 7.5–90, 5–80, 2.5–40, and 2.5–40 μg/mL. Their equations were expressed as Y_IOR_ = 49,524x − 33,321 (*R*^2^ = 0.9973, n = 6), Y_OR_ = 40,430x − 10,848 (*R*^2^ = 0.9965, n = 6), Y_VI_ = 53,732x − 6507.5 (*R*^2^ = 0.9991, n = 6), and Y_IVI_ = 60,522 − 8586.3 (*R*^2^ = 0.9995, n = 6), respectively. The content of orientin, isoorientin, isovitexin, and vitexin in BLER were 0.337%, 0.331%, 0.119%, and 0.186%, respectively.

#### 3.4.2. Static Adsorption and Desorption with Different Macroporous Resins

Before the test, all the resins were pretreated according to the previous reference method [15]. Pretreated macroporous resins (0.5 g) were accurately weighed and added to 20 mL BLER solution (20 mg/mL) in a 100 mL conical flask, respectively. Then, these flasks were shaken at a constant speed (120 rpm) at 30 °C (303 K) for 24 h. Before and after the static adsorption process, the sample solutions (0.5 mL) were taken out and diluted with an equal volume of chromatographic methanol and were fully mixed and filtered through a 0.45 μm nylon membrane for HPLC determination. The adsorption capacity (*Q_e_*, mg/g) of the different flavonoids on the different resins was calculated using Equation (1) as follows:

(1) $$Q_{e}=\frac{(C_{0}-C_{e})V_{i}}{W}$$

where *C*_0_ and *C_e_* are the concentrations (mg/mL) of flavonoids before and after adsorption, *V_i_* (mL) is the volume of the initial sample solution, and *W* (g) is the dry weight of the tested resin.

After the equilibrium adsorption, the resins were filtered and purged with a small quantity of deionized water (<5 mL) twice. After that, they were statically desorbed with 80% ethanol aqueous solution under a constant speed (120 rpm) at 303 K (30 °C) for 24 h. The concentration of the flavonoids in the desorption solution was analyzed, and the desorption capacity (*Q_d_*, mg/g) and the desorption ratio (*D*, %) were calculated using Equations (2) and (3) as follows:

(2) $$Q_{d}=\frac{C_{d}V_{d}}{W}$$

(3) $$D=\frac{Q_{d}}{Q_{e}}\times 100\%$$

where *C_d_* is the concentration of flavonoids in the solution after desorption (mg/mL), and *V_d_* is the volume (mL) of desorption solution, respectively.

#### 3.4.3. Adsorption Isotherms of the Selected Resin

To optimize the adsorption temperature and BLER concentration, the pretreated XAD-7HP (0.5 g) was accurately weighed and introduced into 20 mL BLER solution with different concentrations (10, 15, 20, 25, 30, 40 mg/mL) in a 100 mL conical flask, respectively. Then, these flasks were shaken at a constant speed (120 rpm) at different temperatures (30, 40, 50 °C) for 24 h, respectively. The initial and equilibrium concentrations of the four flavonoids at different temperatures were determined. Moreover, the two well-known theoretical isotherm models were applied to analyze the adsorption correlations between the solute and the resins.

The Langmuir model equation can be expressed as

(4) $$Q_{e}=\frac{Q_{m}C_{e}}{K_{L}+C_{e}}$$

where *Q_e_* (mg/g) and *C_e_* (mg/mL) are the same as mentioned above, *K_L_* is the Langmuir constant, and *Q_m_* (mg/g) is the calculated theoretical maximum adsorption capacity.

The Freundlich model equation can be expressed as

*Q_e_* = *K_F_C_e_*^1/*n*^ (5)

where *Q_e_* (mg/g) and *C_e_* (mL/mg) represent the same as mentioned above, *K_F_* is the Freundlich constant, and 1/n reflects an empirical constant for the adsorption driving force.

#### 3.4.4. Adsorption Kinetics of the Selected Resin

First, 40 mL of BLER solution (20 mg/mL) was added into a stopped conical flask to mix with the pretreated resin (1.0 g). Then, the flask was shaken continuously with a constant speed (120 rpm) at 30 °C (303 K) for 24 h. During the shaking process, 0.5 mL of sample was withdrawn and analyzed at different times from 0 to 24 h. The adsorption kinetics data was further fitted by two classic models, and their equations can be expressed as follows:

The pseudo-first-order model

*ln*(*Q_e_* − *Q_t_*) = *lnQ_e_* − *K*_1_*t* (6)

The pseudo-second-order model

(7) $$\frac{t}{Q_{t}}=\frac{1}{{K_{2}Q_{e}}^{2}}+\frac{t}{Q_{e}}$$

#### 3.4.5. Trial Experiment of Dynamic Adsorption and Desorption on the Selected Resin

First, 5 g of pretreated XAD-7HP resin with bed volume (BV) of 3.5 mL was packed in a miniature glass column for the trial experiment and continuously rinsed with deionized water before use. Then, the 40 BV of BLER solution (20 mg/mL) was passed through the resin with a flow rate of 2 BV/h. The effluent solution from the resin was collected, and the concentrations of the flavonoids were analyzed by HPLC.

After the above adsorption process, the optimum concentration of ethanol desorption was determined by gradient elution mode. Specifically, the resin column was first eluted with deionized water, and then the adsorbed flavonoids were desorbed with different concentrations of ethanol (20%, 40%, 60%, 80%, and 100%) with a solvent volume of 4 BV and a flow rate of 2 BV/h. Afterwards, the collected effluent was subjected to HPLC analysis.

#### 3.4.6. Lab Scale-Up Dynamic Separation

According to the above process of trial dynamic test, 18 g BLER sample solution (3600 mL, 20 mg/mL) was loaded into a glass chromatographic column (2.5 × 40 cm) with 50 g macroporous resin at a speed of 2 BV/h. Then, 4 BV of deionized water, 60% ethanol, and 95% ethanol were applied to elute the resin column, respectively. Finally, the unabsorbed fraction on the resin and the other three eluting fractions were collected and analyzed. The resin-enrichment fractions were applied to validate and compare with the previous trial results. The eluting fractions were then combined to evaporate using a rotary evaporator and freeze-dried.

### 3.5. Purification of CA by HSCCC

#### 3.5.1. HPLC Determination of CA in HSCCC

Although the HPLC method in Section 3.4.1 can be used to determine the content of CA, it is time consuming for analyzing a large number of samples in an HSCCC test. Therefore, a more rapid and easier method of isocratic elution consisting of acetonitrile 0.1% and formic acid/water solution (*v*/*v*, 15:85) was applied, and the other chromatographic conditions were the same as above in Section 3.4.1. The standard curve of CA was Y = 58,789X + 21,307, where Y and X were its concentration (μg/mL) and the value of the peak area, respectively.

#### 3.5.2. Selection of the Two-Phase Solvent Systems

According to the previous references, the different proportion of solvents was applied to form the two-phase solvent system. Then, 2 mL of the upper and lower phase solutions were introduced into a test tube, and the CA sample (1 mg) from the XAD-7HP resin column eluting process was added, shaken thoroughly, and stood for stratification. Then, the content of CA in two-phase solutions was measured by HPLC, and K values were expressed as the peak area of CA in the upper phase divided by that in the lower phase.

#### 3.5.3. Preparation of Two-Phase Solvent System and Sample Solution

The solvent system of ethyl acetate, acetonitrile, acetic acid, and water (7:2:1:10, *v*/*v*) was used for CA purification. These solvents were mixed to equilibrium in the separator funnel overnight, and then the separated upper and lower phases were ultrasonically degassed for 15 min before use. The CA sample (653.6 mg) concentrated from the water eluting fraction of the XAD-7HP resin chromatography was dissolved with 10 mL of the lower phase of the solvent system.

#### 3.5.4. HSCCC Separation for CA

First, the upper phase as stationary phase was filled into the HSCCC coil column. Then, the lower phase as mobile phase was pumped into the column at a flow rate of 2.0 mL/min with a rotation speed of 850 rpm. When the system reached equilibrium, the sample solution was injected through the injection valve. The whole separation experiment was carried out at 30 °C, and the effluent was continuously monitored at a detection wavelength of 325 nm. The interval time for the automatic fraction collector program was set to 5 min in the first 100 min, and changed to 2 min after 100 min. The CA content and purity of effluent collected in the tube were analyzed by HPLC.

## 4. Conclusions

In our study, the performances of six macroporous resins with different properties for the enrichment of IOR, OR, VI, and IVI from BLER were investigated. The XAD-7HP resin exhibited the best adsorption and desorption capacities for the four flavonoids. The static adsorption process of the four flavonoids on XAD-7HP resin fitted well to the Langmuir isotherm model and pseudo-second-order kinetic model. After the dynamic resin column test, the lab scale-up separation indicated that the concentrations of the four flavonoids could produce about 4.5-fold enrichment with acceptable recoveries. In addition, CA was isolated and purified by combining with the dynamic resin separation and HSCCC. Therefore, our study provided some new methods to obtain flavonoids and CA from BLER for food and pharmaceutical applications.

## Figures and Tables

**Table 1:** Physical properties of the tested macroporous resins.

Resin	Molecular Polarity	Particle Size (mm)	Specific Surface Area (m^2^/g)	Average Pore Size (nm)	Moisture (%)
X-5	Non-polar	0.315–1.25	500–600	28–30	67.94
XAD-1180	Non-polar	0.35–0.6	450	40	70.76
AB-8	Weak polar	0.315–1.25	480–520	13–14	70.34
XAD-7HP	Weak polar	0.56–0.71	500	45	70.29
DM301	Mid-polar	0.315–1.25	330–380	14–17	76.58
S-8	Polarity	0.315–1.25	100–120	28–30	68.57

**Table 2:** Langmuir and Freundlich models for the adsorption of BLER flavonoids on XAD-7HP resin.

Flavonoids	Temperature (°C)	Langmuir Equation			Freundlich Equation		
		*K_L_* (mL/g)	*Q_m_* (mg/g)	*R* ^2^	*K_F_* [(mg/g)· (mL/mg) ^1/*n*^)]	1/*n*	*R* ^2^
	30	0.0054	7.5823	0.9952	12.3849	0.2016	0.8570
IOR	40	0.0075	7.2928	0.9987	12.4944	0.2317	0.9294
	50	0.0121	7.2784	0.9979	13.2937	0.2825	0.9271
	30	0.0105	6.9682	0.9933	10.4057	0.3000	0.8585
OR	40	0.0135	6.8032	0.9914	11.2228	0.3529	0.8462
	50	0.0209	6.7483	0.9908	11.8720	0.4272	0.9543
	30	0.0022	1.8115	0.9975	3.0774	0.2124	0.9722
VI	40	0.0020	1.6700	0.9958	2.7055	0.1918	0.9697
	50	0.0020	1.5557	0.9855	2.5031	0.1882	0.9699
	30	0.0040	3.7025	0.9953	6.9508	0.2181	0.9137
IVI	40	0.0037	3.5115	0.9971	6.2293	0.2003	0.8907
	50	0.0047	3.4764	0.9932	6.2998	0.2171	0.8281

**Table 3:** Fitting results of the adsorption kinetics at 30 °C for different flavonoids on the XAD-7HP resin.

Flavonoids	Pseudo-First-Order			Pseudo-Second-Order		
	*K*_1_ (min^−1^)	*Q_e_* (mg/g)	*R* ^2^	*K*_2_ (g/mg·min)	*Q_e_* (mg/g)	*R* ^2^
IOR	3.0757	5.9173	0.9171	0.6644	6.4551	0.9723
OR	2.8931	4.9654	0.9412	0.7260	5.4395	0.9846
VI	4.1682	1.8386	0.9301	3.1501	1.9743	0.9835
IVI	2.3931	2.7024	0.9559	1.0424	2.9988	0.9905

**Table 4:** The K values of CA in different solvent systems.

Solvent System	K
ethyl acetate/ethanol/water (*v*/*v*, 3:2:5)	0.25
ethyl acetate/n-butanol/ethanol/water (*v*/*v*, 3:1:1:5)	0.37
ethyl acetate/n-butanol/water (*v*/*v*, 3:2:5)	0.50
ethyl acetate/n-butanol/water (*v*/*v*, 1:3:4)	0.52
ethyl acetate/acetonitrile/acetic acid/water (*v*/*v*, 7:2:1:10)	0.67

## Data Availability

All data can be found in this paper.

## Supplementary Materials

The following supporting information can be downloaded at: https://www.mdpi.com/article/10.3390/molecules28114443/s1, Figure S1: The molecular structure of the four flavonoids; Figure S2: HPLC chromatograms of mixed standards (A), sample solution (B) and different eluting fractions from XAD-7HP in the scale-up dynamic enrichment experiment: sample loading effluent (C); water eluting fraction (D) and 60% ethanol eluting fraction (E); Figure S3: HSCCC separation chromatograms for CA from XAD-7HP resin; Figure S4: HPLC chromatograms of CA standard (A) and the isolated CA fraction by HSCCC (B).
