# Supplementary material for: Separation of Flavonoids and Purification of Chlorogenic Acid from Bamboo Leaves Extraction Residues by Combination of Macroporous Resin and High-Speed Counter-Current Chromatography
Source: Molecules. 2023 May 30;28(11):4443. doi: 10.3390/molecules28114443 (PMC10254542; doi:10.3390/molecules28114443)
Supplement: Supplementary file 1 [file molecules-28-04443-s001.zip › molecules-2349569-supplementary.pdf]

Supplementary material

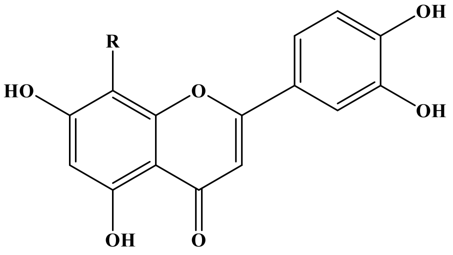

1

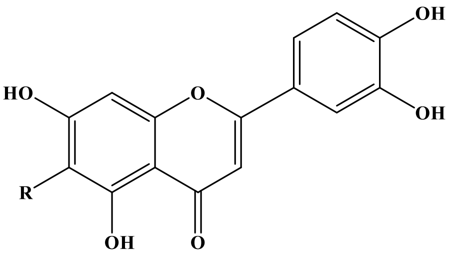

2

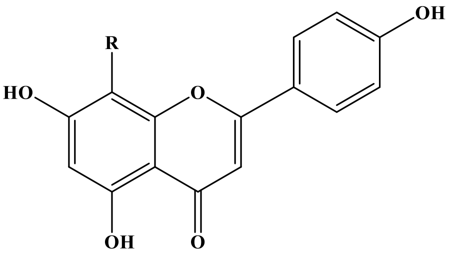

3

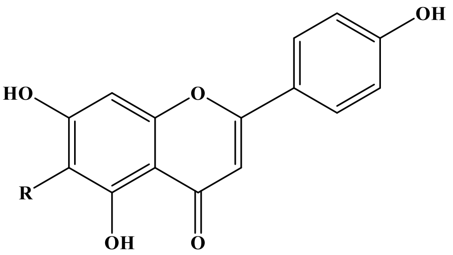

4

Figure S1 The molecular structure of the four flavonoids: 1- orientin (OR), 2- isoorientin (IOR), 3- vitexin (VI), 4- isovitexin (IVI); R= β-D-glucopyranosyl

(A)

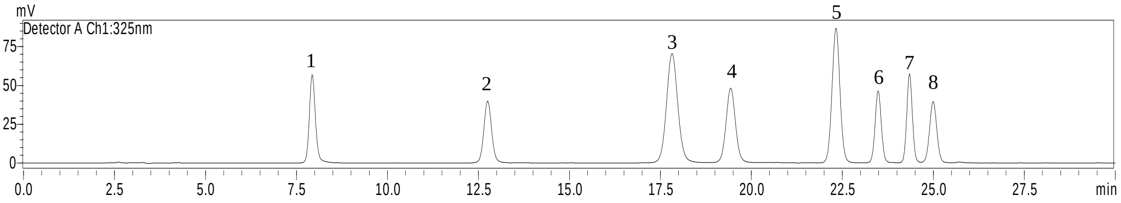

(B)

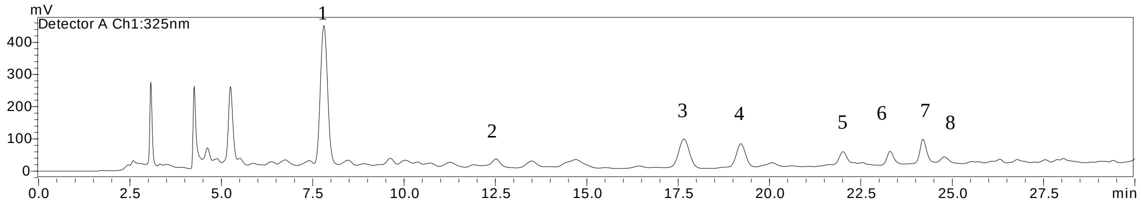

(C)

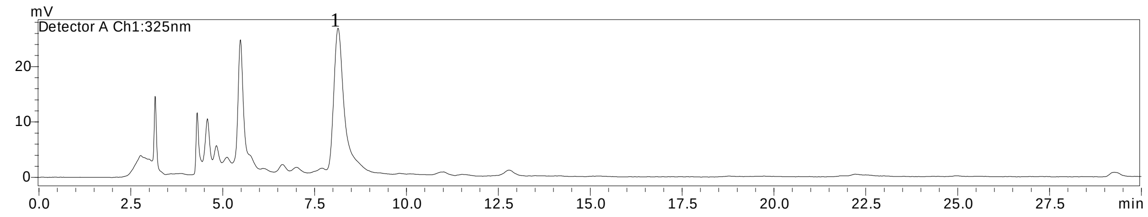

(D)

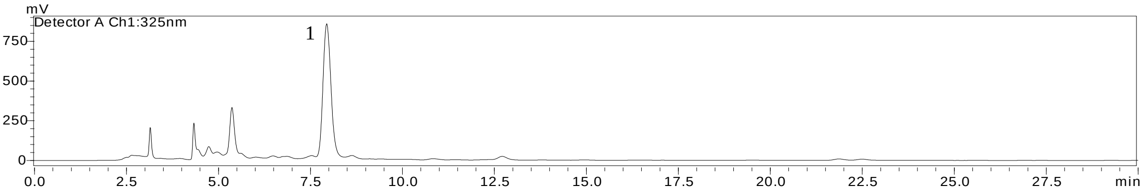

(E)

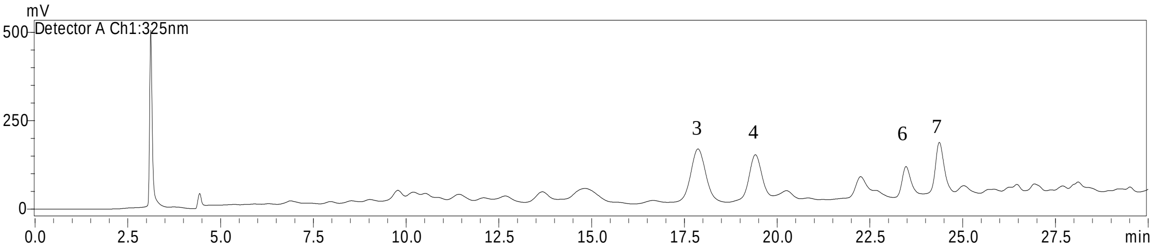

Figure S2 HPLC chromatograms of mixed standards (A), sample solution (B) and different eluting fractions from XAD-7HP in the scale-up dynamic enrichment experiment: sample loading effluent (C); water eluting fraction (D) and 60% ethanol eluting fraction (E).

1- chlorogenic acid (CA), 2- caffeic acid, 3- isoorientin (IOR), 4- orientin (OR), 5- p-counaric acid, 6- vitexin (VI), 7- isovitexin (IVI), 8- ferulic acid

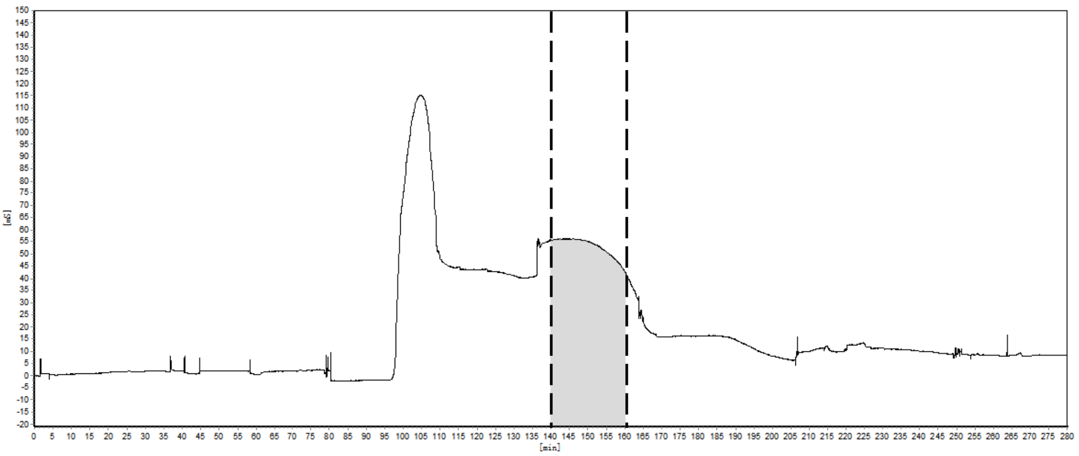

Figure S3 HSCCC separation chromatograms for CA from XAD-7HP resin

(A)

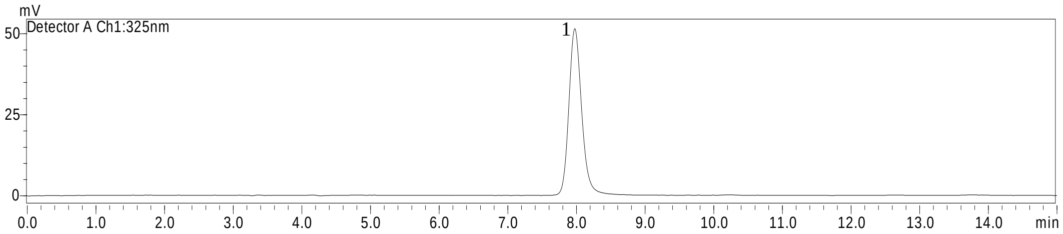

(B)

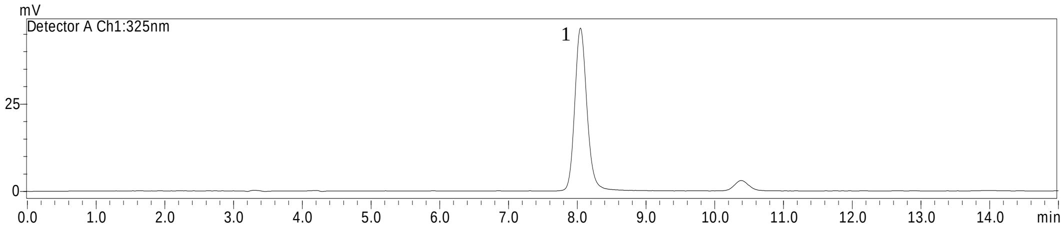

Figure S4 HPLC chromatograms of CA standard (A) and the isolated CA fraction by HSCCC (B). 1- chlorogenic acid (CA)
